# Supporting Older People Living With Frailty to Self‐Manage Multiple Medicines: An Experience‐Based Co‐Design of a Complex Intervention Developed in UK Primary Care

**DOI:** 10.1111/hex.70364

**Published:** 2025-09-07

**Authors:** Giorgia Previdoli, Ruth Simms‐Ellis, Jonathan Silcock, David Phillip Alldred, V‐Lin Cheong, Savi Tyndale‐Biscoe, Justine Tomlinson, Beth Fylan

**Affiliations:** ^1^ Yorkshire Quality and Safety Research Group, Bradford Institute for Health Research Bradford Teaching Hospitals NHS Foundation Trust Bradford UK; ^2^ NIHR Yorkshire and Humber Patient Safety Research Collaboration Bradford UK; ^3^ School of Pharmacy, Optometry and Medical Sciences University of Bradford Bradford UK; ^4^ Department of Health Sciences University of York York UK; ^5^ School of Healthcare University of Leeds Leeds UK; ^6^ Leeds Teaching Hospitals NHS Trust Leeds UK; ^7^ Bradford Teaching Hospitals NHS Foundation Trust Bradford UK

**Keywords:** co‐design, complex intervention development, frailty, medicines self‐management, older people, patient safety, polypharmacy, safe use of medicines

## Abstract

**Background:**

Older people face numerous challenges when managing multiple medicines. They are required to cope with complicated and changing medicines regimens and coordinate input from multiple health and social care professionals. When not well managed, medicines can cause harm, and older people are more susceptible to the impact of errors. Nevertheless, there is a lack of interventions addressing the multiple tasks required for them to manage polypharmacy at home.

**Objective:**

To develop a complex behaviour‐change intervention to support medicines self‐management for older people living with frailty and polypharmacy using Experience‐based Co‐design (EBCD).

**Design:**

EBCD was used to create a prototype of a resilient healthcare‐informed complex intervention with the potential to improve the safety of, and confidence in, medicines self‐management for older people who live at home. Extracts from recordings of interviews about people's experiences of polypharmacy were edited into a short film and shown at meetings to determine priorities. Older people taking 5 or more medicines living with mild‐to‐moderate frailty, their family members and healthcare professionals then participated in co‐design workshops to develop these identified priorities into components of a complex intervention. Two focus groups with healthcare staff, older people, and carers explored potential barriers to implementation.

**Results:**

Shared priorities identified were to support change in the following areas: day‐to‐day practical medicines management; understanding medicines management systems; and communicating with healthcare teams. A logic model was designed to make explicit the intervention's underpinning theory of change. A five‐part complex intervention was developed which addresses behaviours with potential to increase safety in medicines management. Intervention content was mapped to relevant behaviour change techniques to aid clarity, precision and specificity in reporting its characteristics.

**Conclusions:**

Using EBCD we were able to co‐develop a novel support intervention to improve safety in medicines management at home for older people living with frailty which incorporated: (1) knowledge of medicines and checking medicines received; (2) organising medicines supply; (3) adherence and self‐monitoring when taking multiple medicines; (4) dealing with changes in medicines; and (5) knowledge of help available and how, where and when to seek it.

**Patient or Public Contribution:**

A member of the public with lived experience of managing medicines in older age was co‐applicant and co‐author in this study, supported by an advisory group of older people taking multiple medicines or with experience of supporting family members. Their contribution played a key role in shaping a relevant and respectful complex intervention for this population.

## 1 Introduction

As people get older, they become more likely to develop multiple conditions [1] requiring multiple medicines which can cause errors [2]. Studies conducted in different European countries [3, 4] and in the United States [5] reported that between 32% and 44% of people aged 65 or older take at least 5 medicines. Data collected by the English National Health Service (NHS) indicate that 37% of people aged 65 or over took five or more medicines [6], while a study conducted in Scotland found that 35% of people aged 80 or older took 10 or more medicines [7]. Polypharmacy is commonly defined as the use of 5 or more medicines [8]. Going beyond simply counting medicines, the King's Fund differentiates between ‘appropriate polypharmacy’, ‘where medicines use has been optimised and where the medicines are prescribed according to best evidence’ and ‘problematic polypharmacy,’ where ‘the intended benefit of the medication is not realised’ [9 p.9]. In older people, polypharmacy has been linked to a range of negative outcomes, including drug‐related problems, adverse drug events, impact on physical and cognitive function, hospitalisation and mortality [10, 11, 12].

The World Health Organization (WHO) launched a global initiative in 2017 to reduce avoidable harm caused by unsafe practices and errors with medicines [13] and subsequently identified addressing polypharmacy and improving patients' involvement in their care as key priorities [14]. Research has shown that older people face numerous safety risks when managing multiple medicines, especially when complexity of regimen increases [2, 15]. Compared to the ‘robust’ older population living with polypharmacy [16], people living with frailty, an ageing‐related process which increases vulnerability to adverse events [17, 18], are more likely to take 10 or more medicines [19] and receive potentially inappropriate medications [20]. Frailty has also been associated with increased risk of drug to drug interactions, adverse drug reactions, delirium, falls, hospital admissions and institutionalisation [16, 21, 22, 23].

Despite the increased risks, interventions to support polypharmacy management in the frail older population are limited [24, 25, 26]. We know that people living with frailty are generally excluded in clinical trials [22]. Due to lack of evidence [27], assuming that interventions to improve medicines management for the general old population will be suitable or adaptable to people living with frailty might not be appropriate.

Also, managing many medicines at home can be very demanding for older people and those who support them [28]. Nevertheless, the level of work required to manage many medicines, often with differing dosing patterns and formulations (e.g., tablet, patch, liquid, cream, and so on), is under‐recognised by prescribers [29].

There is limited knowledge about how, and by whom, older people living with frailty should be prepared to manage the emotional, relational, practical and safety‐related work required by their complex medicines regimens. However there is evidence from qualitative studies that patients can devise and implement strategies to bolster the safety of how medicines are managed [30], and older patients are able to create their own checklists, ensure timely supplies, solve problems, and seek help to avoid deterioration [31]. As highlighted by the research surrounding resilient healthcare theory, an approach which recognises the need for flexibility to adapt to challenges and disruption to maintain safe conditions [32], patients and families have been overlooked and under‐used as sources of resilience in healthcare systems [33], and this applies to medicines management systems as well [30].

Guidelines to support complex intervention development [34, 35] discuss the importance of involving the people who will use an intervention in all stages of its development. Experienced‐Based Co‐design (EBCD) is a participatory approach that has increasingly been used to embed stakeholders' views in complex intervention development [34, 36, 37]. EBCD has successfully involved patients and staff in the way care is designed and delivered in healthcare settings, such as hospital wards [38, 39, 40]. EBCD has also been adapted to be embedded in multi‐stage medicines safety research studies, for example, to develop a complex intervention to improve safety in medicines management for cardiology patients discharged from hospital [41] and also in a complex intervention to improve the process of deprescribing for older people living with frailty [42].

In this article, we describe how we developed a complex behaviour change intervention informed by resilient healthcare theory—‘I Manage My Meds’— following a partnership approach [34] based on an adaptation of EBCD. This process followed, as a preparatory stage for intervention development, a rapid review of evidence [24] and qualitative interviews with 32 older people living with mild‐moderate frailty and taking five or more medicines [43], and 16 healthcare staff.

## 2 Methods

**Figure 1:**
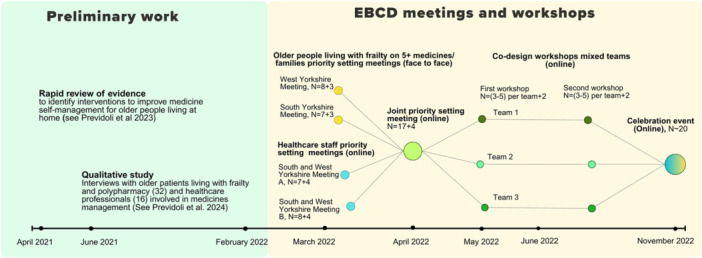
Timeline of the EBCD process.

**Figure 2:**
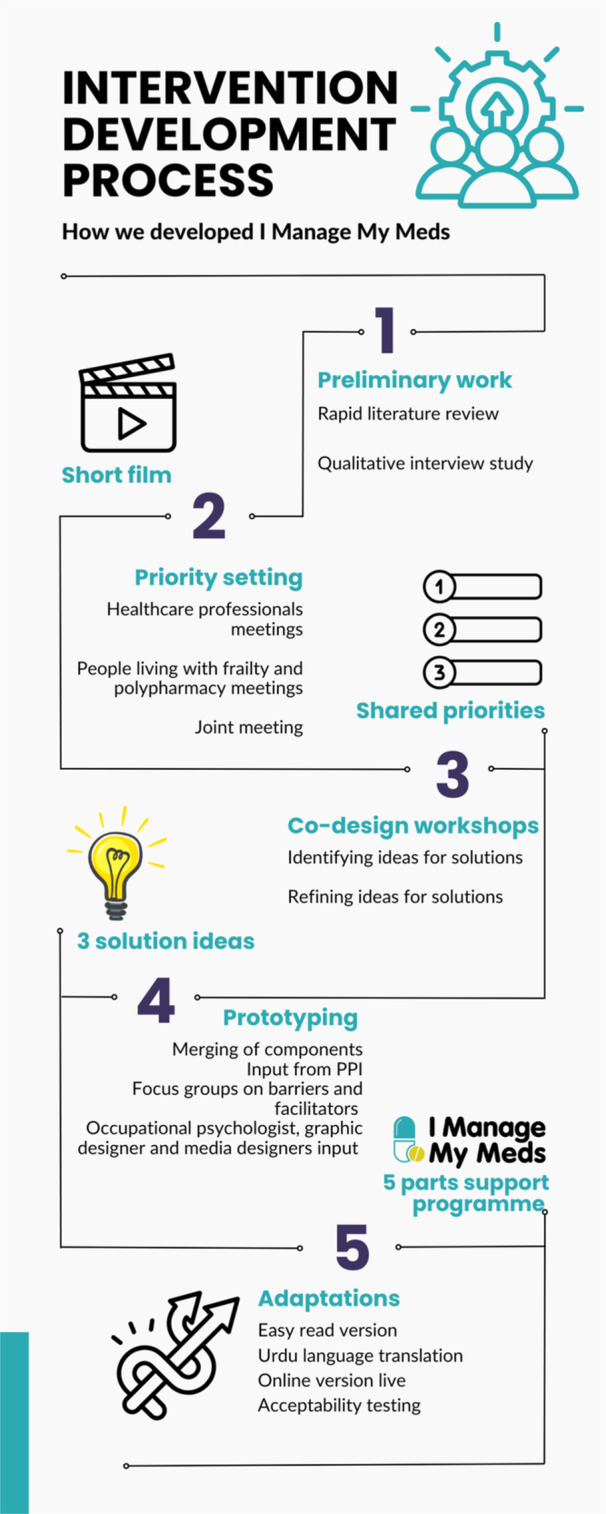
Intervention development process.

A timeline of the adapted EBCD process adopted is reported in Figures 1, and 2 shows how the output of each step in the intervention development process became the starting point of the next. A Guidance for reporting intervention development studies in health research (Guided) checklist [44] was created (Appendix S2) to enhance transparency in reporting intervention development.

### 2.1 Settings and Team

EBCD was conducted with older people living with mild‐moderate frailty and polypharmacy, their family members, and primary and community healthcare staff who support this population with their medicines in South and West Yorkshire, UK, between March and November 2022. Ethical approval for the research components of the study was obtained.

### 2.2 EBCD Adaptation

We embedded EBCD in a multiphase, multi‐site research study to develop an evidence‐based complex intervention, following the methods reported by Fylan and colleagues [36]. EBCD typically comprises three main steps [45]. The first step involves gathering stakeholders' experiences, which usually includes both ethnographic observations and interviews, as well as the preparation of a short film to be used in meetings with stakeholders. The second step involves the identification of problems and setting priorities. The third and final step involves the co‐design of solutions for the agreed priorities. At step one, consistent with other studies [46], we omitted ethnographic observations due to the invasive, resource‐intensive nature of observing people's experience of medicines management in their daily lives at home. Our results from gathering stakeholders' experiences have already been published [43]. In this paper, we focus on the remaining steps in the EBCD process and we describe: (1) Producing a short film of people's experiences of managing their medicines; (2) Stakeholder meetings to identify priorities for change; (3) Co‐design workshops to identify solutions to enhance medicines self‐management; (4) Working with our patient and public involvement (PPI) group and healthcare staff to refine the solutions identified and creating a logic model for the intervention; (5) Collecting feedback from additional stakeholders, developing adaptations and acceptability testing.

### 2.3 EBCD Step One: Producing a Short Film of People's Experiences of Managing Their Medicines

An 18‐min film was produced with edited extracts from recorded interviews with 14 participants. GPr and BF worked together to select emotional touchpoints points in the interviews, as well as examples of adaptive and proactive strategies older people devised to help keep themselves and their medicines management system safe [43], consistent with the resilient healthcare theoretical lens adopted [30, 32, 33].

Clips were selected from the interviews, as the video recordings became available. The film was organised in five chapters: Managing my medicines and my conditions; Routines, reminders and safety nets; Medicines burden, personal adaptations and decision making; Communication with healthcare professionals; Anticipating problems, responding to the unexpected and monitoring what you are doing. Chapter names were chosen in collaboration with the PPI group.

### 2.4 EBCD Step Two: Stakeholder Meetings to Identify Priorities for Change

Three members of the research team (BF, JS, JT) with previous experience of conducting EBCD were involved in the planning and delivering of the meetings and a fourth (GPr) received Point of Care Foundation EBCD training in preparation. In line with the EBCD approach, the short film was used to provoke reflections and conversations at five different stakeholder meetings. The inclusion criteria for stakeholders were: (1) people 65 years and older identified as living with mild to moderate frailty, (electronic Frailty Index [eFI] between 0.12 and 0.36) [47], living at home and who were managing five or more medicines without paid support for medicines administration; (2) family members, or others, supporting them in an unpaid or voluntary capacity; (3) healthcare professionals playing a role in medicines management for older people (GPs, pharmacists, pharmacy technicians, administrative staff, nurses, community healthcare staff).

Older people living with frailty and polypharmacy, family carers and professionals were recruited in two ways. First, all participants in the preliminary interview study [43], were invited to join EBCD by GPr, either by letter or email. Second, participants were recruited by GPr across the National Institute for Health and Care Research (NIHR) Yorkshire and Humber Patient Safety Translational Research Centre (PSTRC) ‘Safety in Numbers’ Group, the Yorkshire and Humber Clinical Research Network and professional networks linked to the research team, either posting via newsletters or by direct email. Older people living with frailty and polypharmacy and professionals were invited by GPr to separate stakeholder meetings first, and then to a joint meeting with mixed stakeholders. GPr provided additional information to participants and answered any questions over the telephone, before the first meeting took place. If they wished, older people were able to join the meeting with someone supporting them.

Two face‐to‐face, 2‐h meetings for people living with frailty and polypharmacy were organised in March 2022, one in Sheffield and one in Leeds. To enable participation transport was organised and paid in advance where possible and a £40 payment was offered to thank participants. BF and GPr facilitated the meetings together, supported by a member of the PPI group. At the start, they introduced an overview of findings from the rapid review [24] and a summary of themes from the interview study [43]. After showing the short film, GPr drew a line with dots on a board, reflecting the chapter structure of the film and representing the emotional touchpoints in the medicines self‐management ‘journey’. Participants were invited to build an ‘emotional map’ by writing down on post‐it notes how their personal experience resonated with the ones in the film and sticking them on the board. Through this process participants identified where along the line they felt changes were most needed. Finally, BF facilitated a discussion aimed to agree a list of priorities for change in medicines self‐management.

Two online priority‐setting meetings with two different groups of healthcare professionals took place in March 2022. We ran those meetings online to accommodate participants' preferences. Both were facilitated by GPr, JT, BF and JS and lasted for 2 h. The meetings began with an overview of findings from the rapid review and early themes from the interview studies, followed by viewing the short film and open discussion on the topics covered by the film. Healthcare staff were then supported to identify priorities for change to improve medicines self‐management at home.

GPr and BF met to revise the emotional maps and the list of properties produced after each meeting. Both were analysed thematically to identify common themes, remove duplicates and merge subthemes (e.g. communication with GP) into more general categories (communication with healthcare professionals).

Everyone involved in priority‐setting meetings was invited to take part in a joint online meeting lasting 2 h. Support was offered to anyone who had never taken part in online meetings. Phone calls for training purpose were arranged before the meeting. First, we guided participants to download and install the videocall app (Zoom) on their device. Then we arranged multiple test video‐calls, to make sure people felt confident using the new system.

To mitigate power dynamics, everyone received the agenda in advance and people living with frailty and polypharmacy received an in‐depth explanation of what to expect in the meetings. We also explained that people knowledge and experience were the essential components of the intervention development process and reassured them that no additional knowledge or preparation was required to take part. The meeting took place in April 2022 and was facilitated by BF, JT, JS and GPr. At the start, the purpose of the research and the process for intervention development were summarised, then the facilitators presented two separate lists of priorities for change, the first identified by people living with frailty and polypharmacy and family members, the second identified by healthcare professionals during the previous meetings. After viewing the short film together, all stakeholders were supported to agree on a shared list of priorities.

### 2.5 EBCD Step Three: Co‐Design Workshops to Identify Solutions to Enhance Medicines Self‐Management

At the end of the joint meeting, we sought participants willing to work in one of three groups—including people living with frailty and polypharmacy, family members and professionals—as a design team. We decided to have three small groups so that the teamwork could be as interactive as possible. Additional older people living with frailty and polypharmacy and family members were invited to join the design teams, to keep the group composition balanced. Participants we approached included members of the study PPI group and patients and carers representatives at the NIHR Yorkshire and Humber PSTRC.

Two professional facilitators (AR and JW) and GPr (as note taker) attended all workshops (two per team, six in total), which took place between May and June 2022. At the first co‐design workshop, the facilitators used a relatable scenario, shopping for food, to stimulate people to talk about what they considered positive or negative experiences in shopping. They then introduced a scenario involving an older person prescribed with multiple medicines for the first time. Participants were encouraged to think of what information and support this person might need to keep safe and organised with her medicines. A collaborative digital whiteboard, ‘Jamboard,’ [48] was used to capture ideas for solutions and organise them thematically. At the end of the first meeting each team agreed one or two ideas to bring forward for refinement. GPr and BF met to prioritise the components of the suggested solutions based on their applicability to medicines safety. At the second co‐design workshop, the notes taken and the ideas for solutions retained were discussed. The two facilitators supported the teams to refine their ideas for solutions, using patient personas (e.g., Melvina, an older woman living with frailty and polypharmacy), medicines management scenarios (e.g., planning to talk to a pharmacist) and capturing ideas on the Jamboard.

### 2.6 Intervention Prototyping

Three ideas for solutions or potential interventions identified by the design groups became the starting point for intervention prototyping.

The research team revised the three ideas for solutions, merged them together and retained only the components aimed at reducing harm from problems with medicines in the frail older population. Findings from a rapid review conducted by the team [24] and the interviews [43] shaped the way ideas were merged and retained, and solutions were shaped into intervention components. For example, the idea that managing many medicines is a skilled and demanding job requiring patients to navigate complex systems [43] guided us to prioritise ideas to make medicines self‐management easier. Our choice of techniques was inspired by those interventions identified by the review, where patients were encouraged to play proactive roles [24]. Iterations of the prototype were first developed by GPr, RSE, BF and then revised by the wider research team and the PPI group in multiple rounds. A graphic designer (AW) created the branding for the prototype, supported by the research team and the patient advisory group, while a media company was involved in the professional filming of ‘video tips’ from older people living with frailty and polypharmacy and pharmacists, following scripts written by GPr, RE, BF and revised by the PPI group and HS. EBCD meetings and workshops were not recorded, but all discussions were noted and then synthetised thematically by GPr and BF. Those summaries contributed to the tone (how participants described their experience) and the content of the scripts (e.g., real‐life examples of issues with communicating medicines changes).

Occupational psychologist RSE helped us to give structure to the intervention prototype. First, using the priorities and intervention content identified by the design groups, we devised clear goals for all elements of the prototype intervention. Second, taking every intervention element in turn, RSE examined systematically each of Michie et al.'s 16 behaviour change technique clusters and their associated individual techniques, to identify our intervention's active components [49]. These were noted.

JS, BF and GPr developed the logic model for intervention [50]. The prototype was then presented at a final EBCD celebration event attended by stakeholders involved in the co‐design in November 2022. Ideas for improvement were collected for future consideration and adaptations.

### 2.7 Collecting Feedback From Additional Stakeholders, Developing Adaptations and Preparing for Acceptability Testing

Between January and March 2023, the prototype was presented at four different events: two in‐person focus groups of older people living with frailty and polypharmacy and family members hosted at two general practices (one in Bradford and one in Leeds), and two online focus groups of healthcare staff involved in medicines management based across West and South Yorkshire. All participants were either living or working in Yorkshire, UK. The two practices which hosted the focus groups were part of ten surgeries sampled in the preliminary study [43]. The first was chosen because they expressed a strong interest in being involved in further steps. The second practice was chosen because it was based in an area with a large Asian or British Asian population, where recruitment in previous stages was hindered by the disproportionate impact of the Covid pandemic [43].

Feedback received informed subsequent iterations. Participants had not previously been involved in the research. Inclusion criteria were: 65 years and older; polypharmacy (5 medicines or more); living with mild to moderate frailty (eFi score between 0.13 and 0.36); living in their own home; manage their own medicines with or without informal support. Staff needed to have a role in medicines management in primary or community care to take part. After database screening the two general practices sent out invitations to attend a focus group to eligible patients. For the healthcare professional focus groups, staff were invited via NHS newsletter and the research team network. Transcripts of meeting recordings and facilitators' notes were analysed deductively to understand barriers and facilitators to implementation. Participant suggestions for improvement were incorporated in the prototype development or reported as alternative options for future refinements. People living with frailty and polypharmacy suggested changing some of the programme's titles and icons (e.g. from ‘forget me not’ to ‘routines and reminders’). They also advised to build each element ‘as stand‐alone’, so people could navigate the programme as they wished, without having to follow a set order. The PPI group then contributed to shaping both content and mode of delivery of the intervention components in the subsequent iterations. For example, they helped the team to decide what type of videos to include in the online prototype. They suggested that we used ‘real people talking’ instead of animation characters, as they would feel more relatable and appropriate by an older audience. Some PPI members volunteered to be filmed for the prototype.

## 3 Results

### 3.1 Participants and Settings

Table 1 reports numbers and types of stakeholders involved in each EBCD step.

**Table 1:** Experience‐based co‐design participants at each meeting.

EBCD Step	Type of meeting	Type of stakeholders and numbers
Priority‐setting meeting for older people living with frailty and polypharmacy and families	Two face‐to‐face meetings	18 people: 12 older people living with frailty and polypharmacy, three family members, two facilitators and one note taker.

Priority‐setting meeting for healthcare professionals	Two online meetings	19 people: 15 healthcare professionals (practice pharmacists, practice pharmacist technicians, practice nurse, community healthcare staff), four facilitators.

Shared priorities—Joint Meeting	One online meeting	21 people: seven older people living with frailty and polypharmacy, one family member, nine healthcare professionals (pharmacists, pharmacy technicians and community matron), four facilitators.
Co‐design workshops	Six online workshops	19 people: seven older people living with frailty and polypharmacy, two family members, seven healthcare professionals (pharmacy technician and practice pharmacist), two facilitators and one note taker.
Celebration event	One online meeting	Over 20 people took part, including older people living with frailty and polypharmacy involved in the research who could not join the co‐design team. Attendance was not formally recorded.

### 3.2 EBCD Step Two: Priority‐Setting Meetings

In their meetings, older people living with frailty and their families talked about the challenges of managing multiple medicines in a system that is difficult to navigate, where preparation is limited, the confusion caused by medicines often changing appearance and the difficulties of identifying support available. Participants found many similarities between the themes touched upon by the short film and their own experiences. A merged list of priorities agreed at those meetings is reported in Table 2: In their meetings, healthcare professionals shared their experiences of supporting older frail people taking multiple medicines and, after watching the short film, they discussed the challenges of providing person‐centred support in an overstretched system. A merged list of priorities agreed at those meetings is reported in Table 2. Subsequently, healthcare staff, older people living with frailty and polypharmacy and family members met and worked together to discuss and agree what shared priorities to bring forward. The final joint list of priorities agreed by participants is reported in Table 2.

**Table 2:** Priorities for change identified by each group.

Older people living with frailty and polypharmacy and families	Healthcare professionals	Joint
1. Get organised and set up routines when medicines are started. 2. Anticipate common problems and mistakes and know how to respond. 3. Detect warning signs (e.g., adverse reactions) and identify help. 4. Deal with frequent changes and their impact on your life.	1. Support patients in maintaining ownership of their medicines. 2. Reduce burden of treatment. 3. Remove barriers to communication with patients (e.g., language, digital exclusion). 4. Support patients to get organised and establish systems and routines. 5. Support patients in navigating the system.	1. Day‐to‐day practical management: learning to live with my medicines. 2. Understanding and navigating the wider medicines management system. 3. Communication with the healthcare team—finding the answers to my questions about my medicines.

### 3.3 EBCD Step Three: Co‐design Workshops

Participants formed three groups, each comprising 3–6 people with a balance of older people living with frailty and polypharmacy/family and healthcare professionals. After discussion and voting, five solutions were chosen to be brought forward. At the second round of workshops, the groups refined three retained solutions. These are in Table 3. In addition to solutions, the groups identified three key principles for any future development, reported in Table 4.

**Table 3:** Principles followed in intervention development.

1	Maximise inclusivity, for example, by providing multiple ways to access content through face‐to‐face, online, video, and printed materials.
2	Acknowledge and celebrate personal preferences, by giving people options to choose what works best for them.
3	Put people and experiences at the centre, by adopting the patient's perspective (e.g., having real patients sharing tips, rather than professionals telling people what to do).

**Table 4:** Ideas for solutions.

1	Quick‐start guide for patients new to multiple medicines:
	Testing out my new routine plans and adjusting them to make them easier. Building new routines around my usual activities and managing emergencies. Preparing for a review. Learning modules about the systems I will encounter in my new meds management life. Who does what for me and when. Learning to be assertive when discussing my meds.
2	Tips and tricks for me and my meds:
	Multi‐module resources that help me share and see other's ideas for day‐to‐day management.
	‘How to’ guides, such as getting my repeat medicines. Educational and problem‐solving tools (apps and digital as well as paper and other reminders, checklists and charts). Emergency contacts and planning tools for holidays, reviews. How to use your preferred ways to communicate with the right bit of the system and use GP systems to help custom requests.
3	My meds mate:
	Preparing for a review as two‐way communication.
	Checklists and audio‐visual material to help me list my questions—what to think about and what to ask. Tackling difficult questions with my healthcare professional (HCP) Assertiveness—practising and rehearsing. HCPs sharing their requirements for a review, so I understand why they ask me certain questions. Keeping updated after the review—notify me of any changes in medicines and next steps.

### 3.4 Prototyping: Intervention and Logic Model Development

The final prototype comprised a five‐part support programme ‘I Manage My Meds’, which is available online with an offline face‐to‐face version and facilitator guide. The intervention is organised into five sections: Always check what you get; Keep on top of your supply; Routines and reminders; Changes to watch out for; and Time to ask for help. It comprises short patient‐led videos with tips and suggestions for self‐management techniques, and downloadable handouts. In total, 11 different behaviour change techniques are active within the intervention:

1. Shaping knowledge
2. Social support
3. Comparison of behaviour
4. Self belief
5. Goals and planning
6. Natural consequences
7. Associations
8. Feedback and monitoring
9. Regulation
10. Antecedents
11. Repetition and substitution.

These are detailed by module in Table 5.

**Table 5:** High‐level view of the five‐module ‘I Manage My Meds’ programme.

Module 1: Always check what you get	
**Content and goals**	**Behaviour change techniques involved**
Why you need to know exactly what medicines you take and why, and that they are correct when you get them from the pharmacy.	**Repetition and substitution:** Behavioural rehearsal/practice. **Natural Consequences:** Health consequences; Salience of consequences. **Goals and planning:** Goal setting (behaviour); Problem solving/coping planning; Action planning. **Social support:** General and practical. **Comparison of behaviour:** Modelling of the behaviour; Social comparison. **Self‐belief:** Verbal persuasion to boost self‐efficacy. **Shaping knowledge:** Instructions on how to perform the behaviour.
Helps you with:	
1. Keeping and updating a log of your medicines. 2. Checking your medicines are right using a step‐by‐step process. 3. Getting help if something isn't right.	

Module 2: Keep on top of your supply	
**Content and Goals**	**Behaviour change techniques involved**
How best to order your medicines from the GP, how to avoid running out and storing everything safely.	**Associations:** Prompts/cues. **Antecedents:** Restructuring the physical environment; Changing exposure to cues for the behaviour. **Natural Consequences:** Health consequences; Salience of consequences; Social & environment consequences. **Goals and planning:** Goal setting (behaviour); Problem solving/coping planning; Action planning. **Social support:** General and practical. **Comparison of behaviour:** Modelling of the behaviour; Social comparison. **Self‐belief:** Verbal persuasion to boost self‐efficacy. **Shaping knowledge:** Instructions on how to perform the behaviour.
Helps you with:	
1. The different ways you can order medicines. 2. How to avoid running out of medicines. 3. Ensuring you have enough medicines when your routines change (e.g., going on holiday). 4. Storing your medicines safely.	

Module 3: Routines and reminders	
**Content and goals**	**Behaviour change techniques involved**
Tips for taking the right medicine at the right time to boost your memory and confidence.	**Associations:** Prompts/cues. **Antecedents:** Restructuring the physical environment; Changing exposure to cues for the behaviour. **Natural Consequences:** Health consequences; Salience of consequences. **Goals and planning:** Goal setting (behaviour); Problem solving/coping planning; Action planning. **Social support:** General and practical. **Comparison of behaviour:** Modelling of the behaviour; Social comparison. **Self‐belief:** Verbal persuasion to boost self‐efficacy. **Shaping knowledge:** Instructions on how to perform the behaviour.
Helps you with:	
1. Setting up your own system to take the right medicine at the right time. 2. Tips for checking if you've taken your medicines.	

Module 4: Changes to watch out for	
**Content and goals**	**Behaviour change techniques involved**
Simple tips to help minimise any problems and disruptions when your medicines are changed (e.g., after a hospital stay or specialist appointment). Helps you with: 1. When you might need to take extra care and why. 2. Stopping mistakes from happening when you're more at risk.	**Natural Consequences:** Health consequences; Salience of consequences. **Goals and planning:** Goal setting (behaviour); Problem solving/coping planning; Action planning. **Social support:** General and practical. **Comparison of behaviour:** Modelling of the behaviour; Social comparison. **Self‐belief:** Verbal persuasion to boost self‐efficacy. **Shaping knowledge:** Instructions on how to perform the behaviour.

Module 5: Time to ask for help	
**Content and goals**	**Behaviour change techniques involved**
What to do if you feel ‘off’, overwhelmed or are struggling to do your daily activities.	**Associations:** Prompts/cues. **Natural Consequences:** Health consequences; Salience of consequences. **Feedback and monitoring:** Self‐monitoring of behaviour. **Goals and planning:** Goal setting (behaviour); Problem solving/coping planning; Action planning. **Social support:** General and practical. **Comparison of behaviour:** Modelling of the behaviour; Social comparison. **Regulation:** Pharmacological support; Regulate negative emotions. **Self‐belief:** Verbal persuasion to boost self‐efficacy. **Shaping knowledge:** Instructions on how to perform the behaviour.
Helps you with:	
1. Monitoring how you feel every day. 2. Spotting key signs that something might not be right with your medicines. 3. Picking the best service to help you, based on how you feel. 4. Reaching out for help if you're overwhelmed or struggling to manage your medicines	

**Figure 3:**
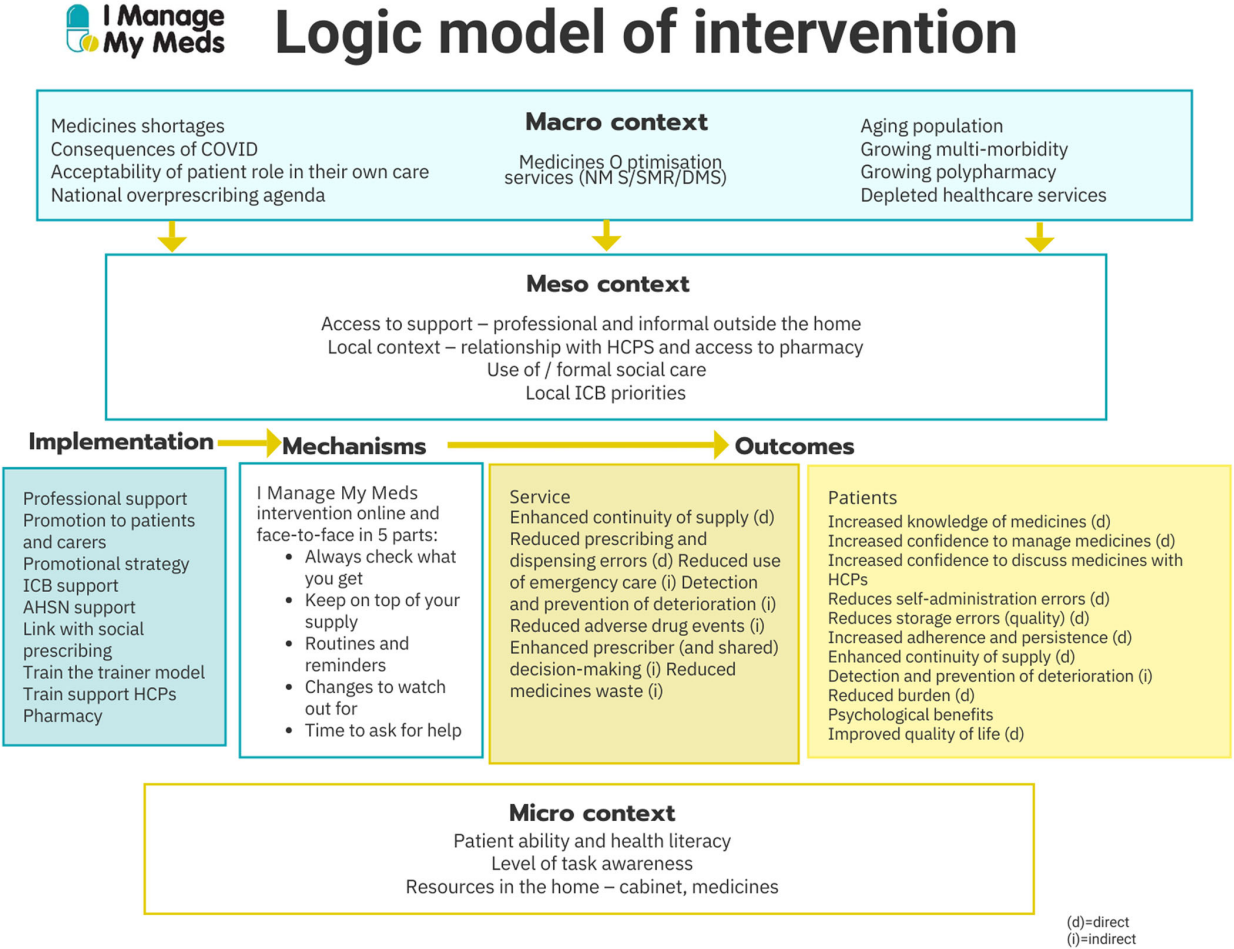
Logic model of intervention.

A facilitator guide was developed for a face‐to‐face version. The full intervention is available online at https://imanagemymeds.org/ and a comprehensive TIDiER (template for intervention description and replication) compliant description is provided in Appendix S1, while the process followed in intervention development is reported in Appendix S2. A real‐world logic model for the intervention was developed [50] and is presented in Figure 3. Table 5 details the behaviour change techniques [49] underpinning the intervention.

### 3.5 Feedback From Additional Stakeholders Regarding Acceptability

Two staff focus groups (online) took place in January 2023 and two older people living with frailty and polypharmacy focus groups (face‐to‐face) were held in February 2023. Eight healthcare staff consented to take part (four in each group) including pharmacists, pharmacy technicians, and reception staff. Fifteen people in total took part; 11 attended the first group and five took part in the second group.

Participants discussed barriers and facilitators to intervention implementation. Both older people living with frailty and polypharmacy and staff expressed how the programme covered essential aspects of medicines self‐management that they felt were relevant and especially needed by people just started on polypharmacy. They liked how the content was delivered from a patient's perspective, which was considered important for engagement and motivation, and that it was non‐judgemental, supporting individuals to choose what works for them and reinforcing their sense of ownership in relation to their medicines. Barriers included staff resources for delivering the face‐to‐face version and for following‐up.

## 4 Discussion

Previous research highlighted that people living with frailty and polypharmacy encounter specific challenges [16] and face potentially worse consequences if things with their medicines go wrong [21, 23]. Nevertheless, interventions targeting this population are scarce [24, 25, 26] and mostly consist of pharmacist‐led deprescribing [51] and medications reviews [52]. This study contributes to this gap in the literature by specifically focusing on older people living with frailty and polypharmacy and examining both the under‐recognised roles [33] patients play in medicines management systems [30], and the complex skill set [43] required in the day‐to‐day management of polypharmacy at home.

In this paper, we presented the participatory approach followed to develop a complex intervention to enhance experiences of medicines self‐management and improve safety for older people living with frailty and polypharmacy. EBCD was chosen because of the powerful role people's experiences can play in improving care [36]. Members of our research team had also successfully adopted EBCD to develop complex medicines safety interventions with similar populations [42] and as part of multisite research study [41].

Older people living with frailty and polypharmacy and healthcare professional priorities highlighted the need to provide enhanced support for the day‐to‐day complicated job of managing multiple medicines. Co‐design workshops led to the development of a set of public‐facing materials organised around five key skill areas: checking the accuracy of supplied medicines; managing supplies, including ordering and storage; developing routines to embed medicines regimens into daily life; managing changes to medicines and medicines routines; and knowing when and how to seek help with medicines. Recent consensus research with healthcare professionals has identified the lack of tools for healthcare providers to support patients with medicines self‐management [53]. This co‐design study has filled that gap and provided important tools that may effectively drive safer medicines self‐management.

### 4.1 Prescribing Medicines, Prescribing Work

Our intervention addressed the increasing number and complexity of medicines prescribed to older people. In the UK, medicines are the second highest cost to the NHS [54] and managing polypharmacy can be burdensome to older people and their family carers. Increasing levels of multi‐morbidity have led to spiralling levels of prescribing to the older population and lately increasing concern about the impact of problematic polypharmacy and overprescribing [55]. The qualitative study that informed this intervention showed how medicines usage could affect people's quality of life, becoming a central aspect of daily activities, and leading some to stop or alter their regimens without consulting their prescribers [43]. This was also found in a recent USA study [56].

Previous research has shown that patients were able to conceptualise skills and responsibilities involved in safe medicines management [57]. Co‐design participants in our study were able to make suggestions to improve how people could cope with the work generated by polypharmacy and importantly to prepare for conversations with healthcare professionals when they may feel overwhelmed. Importantly and in line with UK priorities around over‐prescribing, the co‐designed materials can support conversations about simplifying medicines regimens, reducing the number of medicines where possible, rationalising doses, and discontinuing medicines that are no longer necessary. Polypharmacy can gradually build over time and become overwhelming in its demands on older people and their families. Recent policy [6] has highlighted how both system and cultural change is needed to reduce over‐reliance on medicines.

In making decisions to prescribe polypharmacy, prescribers are also increasing the medicines self‐management burden for their patients. For many, the burden is manageable with support. For others, for example, people living with dementia or cognitive impairments, with lower levels of health literacy or language support needs, other types of intervention to manage polypharmacy will be necessary. The materials developed during this study will not necessarily be usable by every person prescribed multiple medicines. However, adaptations are possible and we have already completed easy‐read and translations of intervention materials.

The use of co‐production in applied health research has increased dramatically in Western countries in recent years, also in response to changing culture in funding institutions [58]. Critical approaches have highlighted the risk of nominal use of PPI, lacking real participation. Co‐design and reciprocity meet several limitations in healthcare settings and ethical implications of patient's involvement must be taken into account [59]. During meeting discussions, for example, many older people reported frustration about their medicines changing brand and appearance, while healthcare professionals worried that people could become over reliant on the look of their medicines. On the one hand, the EBCD process allowed older people to explain why changes in brand felt upsetting; on the other, it allowed healthcare professionals to explain why changes were sometimes unavoidable (e.g., global shortage). Building a mutual understanding offered the two groups the opportunity to identify improvements workable for both. Guidelines to report co‐design studies used to develop complex health interventions are still under development [60]. In the absence of such guidelines, we have presented detailed descriptions of each step and the contribution made by different stakeholders in developing the 'I Manage My Meds' intervention and the work required to achieve such levels of involvement, whilst following a structured research‐driven process. Research describing interventions has been accused of limited success in fully reporting how collective actions were taken and decisions made in intervention development [61]. In this article, we drew upon an established taxonomy of behaviour change techniques to describe our intervention with precision and specificity. We also included a GUIDED checklist [44] to report how the intervention was developed (Appendix S2) and a TiDier Checklist [62] to describe intervention components and planned delivery mode (Appendix S1) to enhance transparency, reduce research waste and support implementation.

### 4.2 Positionality Statement

We acknowledge our positions as applied health researchers, some with professional backgrounds in healthcare and behavioural science, which shaped our thinking about medicines safety. We worked closely with older people and families, valuing their lived experience and ensuring their voices directed the co‐design process. This helped us remain grounded in the realities of medicines self‐management, challenging our assumptions and enhancing the relevance of the intervention.

### 4.3 Limitations

This study used EBCD to develop an intervention as part of a multi‐stage research study [36]. The Point of Care Foundation stresses the importance of stability in the participant group engaged in each EBCD stage. Our study involved older people living with frailty and polypharmacy from different areas of Yorkshire and not every participant was able to attend every meeting. The COVID‐19 pandemic also disrupted some processes, requiring meetings to take place online rather than in person. Whilst the research team offered support to participants to join online meetings, three people did not want to do so, and the experience of online meetings is undoubtedly different to those that take place in person.

We drew upon Behaviour Change Techniques taxonomy [49] to aid description and clarity of our EBCD intervention. Readers interested in deeper conceptual integration between ‘Behaviour Change Wheel’ theory [63] and EBCD for intervention development can access guidance within a published study [64].

Due to time and funding constraints, the intervention prototyping intervention was led by the research team, supported by the PPI group and feedback from additional stakeholders.

### 4.4 Future Direction

Following further development of the prototype intervention, an acceptability questionnaire has been developed based on the Theoretical Framework of Acceptability (TFA) [65]. An acceptability study will seek to understand if the intervention can support older people living with frailty from different backgrounds in managing polypharmacy. Two additional versions of the online intervention have been developed: an easy‐read and an Urdu translation. Input from several PPIE groups, including an older people's forum and a local South Asian network, was sought to develop these adaptations. A hard‐copy version of the intervention is also in development which includes QR codes to access online video resources.

This intervention might be suitable for the wider older population living with polypharmacy. Additional acceptability tests should be considered to determine this.

## Author Contributions

Giorgia Previdoli drafted the paper with support from Beth Fylan and Ruth Simms‐Ellis. Beth Fylan and Savi Tyndale‐Biscoe had the initial idea for this study. Beth Fylan led the bid application and was the project's principal investigator. Giorgia Previdoli and Ruth Simms‐Ellis managed and delivered the development of the intervention. Jon Silcock, David Alldred, V‐Lin Cheong and Savi Tyndale‐Biscoe supported the project delivery and were involved in the conception (by contributing to the original grant application) and ongoing design of the study. Justine Tomlinson, Beth Fylan and Jon Silcock co‐led with Giorgia Previdoli the priority setting meetings with patients and staff. The whole team contributed to the review and refinement of the intervention's components. All authors reviewed, edited and approved the manuscript.

## Ethics Statement

Ethical approval for the research components of the study was obtained (IRAS ID: 282508, REC reference number: 21/SC/0116).

## Conflicts of Interest

The authors declare no conflicts of interest.

## Supporting information

Appendix S1: TIDiER Checklist.

Appendis S2: Guided Checklist to report intervention development.

## Data Availability

Research data are not shared for reasons associated with confidentiality and protection of human privacy.
