# Supplementary material for: Supporting Older People Living With Frailty to Self‐Manage Multiple Medicines: An Experience‐Based Co‐Design of a Complex Intervention Developed in UK Primary Care
Source: Health Expect. 2025 Sep 7;28(5):e70364. doi: 10.1111/hex.70364 (PMC12415351; doi:10.1111/hex.70364)
Supplement: Supplementary file 1 — Appendix S1: TIDiER Checklist. [file HEX-28-e70364-s002.docx]

**Appendix 1 TIDiER Checklist**

Adapted from Hoffmann T C, Glasziou P P, Boutron I, Milne R, Perera R, Moher D et al. Better reporting of interventions: template for intervention description and replication (TIDieR) checklist and guide BMJ 2014; 348 :g1687 doi:10.1136/bmj.g16

| **BRIEF NAME:** | **I Manage My Meds. A five part support programme to help you manage your medicines safely and with confidence.** |
| --- | --- |
| **WHY**  *Describe any rationale, theory, or goal of the elements essential to the intervention. (this could also be the revised introduction of the paper)* | The World Health Organisation identified medicines errors and medicines related bad practices as major causes of avoidable harm. Polypharmacy, the use of 5 or more medicines, has been recognised as a key area to address to reduce harm from medicines. In older age, managing many medicines is particularly demanding and complex; frailty makes health outcomes worse if patients experience problems or errors with medicines. Very few interventions have targeted older patients with polypharmacy and we found only one which was for frail older patients. Most seek to improve adherence only. Our interviews with elderly frail patients taught us that managing multiple medicines felt like a complex job, with limited preparation and support from various, often confusing, overlapping healthcare roles. We used Experience Based Co-Design (EBCD) to build a complex intervention to support patients aged 65 or older, about to start taking 5 or more medicines, to self-manage their medicines safely and confidently. EBCD is a participatory method which helps to increase the relevance and acceptability of intervention content and format. By also involving staff in the co-design we ensured that the complex intervention has potential to be developed in primary care despite the multiple constrains and pressures currently experienced in the UK national healthcare system. We adopted a resilient healthcare approach and drew upon behaviour change theory. |
| **WHAT**  *Materials: Describe any physical or informational materials used in the intervention, including those provided to participants or used in intervention delivery or in training of intervention providers. Provide information on where the materials can be accessed (e.g. online appendix, URL). Procedures: Describe each of the procedures, activities, and/or processes used in the intervention, including any enabling or support activities* | **I Manage My Meds Online Programme**  This is an online toolkit that people can freely access from a public website, <https://imanagemymeds.org> organised in 5 parts:   1. Always check what you get 2. Keep on top of your supply 3. Routines and reminders 4. Changes to watch out for 5. Time to ask for help   Each part includes:   - Patient led videos (1 to 3 mins) describing a range of strategies that patients can try to improve confidence and safely. - Patient led videos describing step by step processes (e.g. how to check medicines received) and how to use the tools provided on the platform (e.g. medicines list) - Tools to download and print (e.g. medicines chart, useful questions when medicines are changed) - Key messages handouts (one for each part) to download and print - Patient led video summarising key messages covered in each part - Links to external trusted resources (e.g. NHS website) - Text and images explaining preliminary or subsidiary information in a clear and concise way, like for example:   - where to find key information about medicines   - how to identify problems with medicines   - what to do when problems arise (scenarios)   On the platform is also available a downloadable diary-style document where users can make notes of what they intend to try.  **I Manage My Meds Patient Support Groups**  This is a half day long support programme organised in 5 parts, run by an expert patient previously trained, supported by a practice pharmacist or pharmacy technician:   1. Always check what you get 2. Keep on top of your supply 3. Routines and reminders 4. Changes to watch out for 5. Time to ask for help   We produced a facilitator guide to enable trained expert patients to set up and manage medicines self-management group sessions. The sessions are aimed at older patients who are about to start 5 or more medicines. The sessions could be run at the patient’s GP practice or at suitable venue in the community, to accommodate subgroups’ preferences. The programme described in the facilitator guide includes a variety of activities and resources. Participants:   - Will have access to patient led videos hosted on the I Manage My Meds Online platform - Will receive printed copies of tools, also available on I Manage My Meds Online platform (medicines list, medicines chart, routine builder, question to ask etc) - Will receive easy read version of key messages for each programme part - Will receive a diary stile document where they can note the strategies/tools they intend to try - Will take part in group discussion - Will take part in simulation activities, for example running a step by step check to identify problems with a fake pack of medicines received - Will have the opportunity to ask questions to a pharmacist or pharmacy technician at the end of the session.   **Preliminary activities:**  Minimum training requirement to become a patient expert facilitator is having completed the I Manage My Meds Online programme. Additional requirements might be specified by the GP practice/organisation hosting the programme and may be adapted to subgroups’ needs and preferences.  No preliminary activities are required to join the online I Manage My Medicines programme. |
| **WHO PROVIDED** | I Manage My Meds Online will be accessed directly by patients and or their family members. Part 5: Time to ask for help, is partially provided by pharmacists with specialist experience in polypharmacy.  I Manage My Meds Support Groups are run by trained expert patients supported by a member of the GP practice, most likely a pharmacist or a pharmacy technician. |
| **HOW**  *Describe the modes of delivery (e.g. face-to-face or by some other mechanism, such as internet or telephone) of the intervention and whether it was provided individually or in a group.* | I Manage My Meds online will be accessed directly by individuals who have either a pc, a tablet or a smart phone and an internet connection. The link to the online platform will be given to them by members of their healthcare team, for example after a structured medications review or after an appointment where multiple new medicines have been prescribed.  I manage my meds support groups is going to be run as group session delivered in the community. Patients (alone or with a family member/friend if they prefer) will be invited to join a support group session and will receive a written invitation by a member of their healthcare team. |
| **WHERE**  *Describe the type(s) of location(s) where the intervention occurred, including any necessary infrastructure or relevant features.* | I manage my meds support groups will be delivered at GP practices and at any additional community venue meeting the needs and preferences of patients’ subgroups. The room will be set up with a projector, an internet connection, table and chairs. |
| **WHEN AND HOW MUCH**  *Describe the number of times the intervention was delivered and over what period of time including the number of sessions, their schedule, and their duration, intensity or dose.* | Feedback from our Patient and Public Involvement group was that completing the online version of I Manage My Meds from the start to the end can require up to 90 minutes.  All parts and subparts have been structured to be self-contained, following feedback received during the patients and staff focus group: people value being able to choose and do only the parts they find most relevant and interesting for their specific needs. I Manage My Meds Online give the users the options of both repeating parts and skipping sessions if they chose so.  I Manage My Meds Support group sessions have an estimated length of two hours and thirty minutes, if all content is covered from start to end, and was designed to be run on one day only. The facilitator handbook gives indications about possible variations in the timing for the activities, if the groups require more time or the GP practice prefers to run multiple shorter sessions. |
| **TAILORING**  *If the intervention was planned to be personalised, titrated or adapted, then describe what, why, when, and how.* | Tailoring has been embedded into the modular intervention. As discussed earlier, those accessing the intervention online can choose to do aspects that are most relevant to them. GP practices delivering the face-to-face version of the intervention also have capacity to break this into separate smaller support groups and involve appropriate patients and families. |
| **MODIFICATIONS**  *If the intervention was modified during the course of the study, describe the changes (what, why, when, and how).* | The intervention still needs to be tested and implemented. The prototyping stage included multiple iterations. At each feedback from different stakeholders and evidence that became available at the time was embedded and informed subsequent iteration.  Adaptations of both the online intervention have been developed, working with communities and people from minoritised groups around Yorkshire and Humber. Collaborations with an existing regional network supporting involvement of people from diverse ethnic backgrounds have been established.  Reasons why adaptations are needed:  A limited number of patients from diverse backgrounds were included in the patient qualitative research study. In addition, ethnic background of participants involved in the interventions reviewed from the literature was not always reported. Information about the ethnicity of staff involved in the interview study and of staff and patients involved in the co-design stage was not collected. Despite limited availability of published evidence, a recent review highlighted the unmet needs around communication and healthcare literacy encountered by older patients with polypharmacy from minoritised groups. A focus group conducted with a group of Asian British (Pakistani) patients and family members revealed language barriers and the importance of families’ and friends’ involvement in medicines management. Our adaptations aim to address these unmet needs, working in partnership with groups and communities, to ensure our intervention is inclusive and does not exacerbate existing health disparities. |
| **HOW WELL**  *Planned: If intervention adherence or fidelity was assessed, describe how and by whom, and if any strategies were used to maintain or improve fidelity, describe them.*  *Actual: If intervention adherence or fidelity was assessed, describe the extent to which the intervention was delivered as planned.* | Not explored yet, for future development. |
