# Supplementary material for: Supporting Older People Living With Frailty to Self‐Manage Multiple Medicines: An Experience‐Based Co‐Design of a Complex Intervention Developed in UK Primary Care
Source: Health Expect. 2025 Sep 7;28(5):e70364. doi: 10.1111/hex.70364 (PMC12415351; doi:10.1111/hex.70364)
Supplement: Supplementary file 2 — Appendis S2: Guided Checklist to report intervention development. [file HEX-28-e70364-s001.docx]

**Appendix 2 GUIDED Checklist**

Duncan E, O'Cathain A, Rousseau N, et al. Guidance for reporting intervention development studies in health research (GUIDED): an evidence-based consensus study. BMJ Open 2020;10:e033516. doi:10.1136/ bmjopen-2019-033516

| **Item 1**  **Description:** Context for which the intervention was developed  **Explanation:** Understanding the context in which an intervention was developed informs readers about the suitability and transferability of the intervention to the context in which they are considering evaluating, adapting or using the intervention. Context here can include place and organisational and wider sociopolitical factors that may influence the development and/or delivery of the intervention | The World Health Organisation (WHO) identified medicines errors and medicines related bad practices as major causes of avoidable harm. Polypharmacy, the use of 5 or more medicines, has been recognised as key area to address to reduce harm from medicines. Observational studies have shown that in older age, managing many medicines is particularly demanding and complex. Research has highlighted that age and frailty contribute to making health outcomes worse, if patients experience problems or errors with medicines.  Adopting a resilient healthcare approach, we conducted a qualitative study in the North of England, UK, to find out about older frail patients' experiences of medicines self-management (Previdoli et al. 2024). We intended to learn from what they already do to keep safe around their medicines and how healthcare staff support them with that. We also conducted a rapid review of interventions (Previdoli et al. 2023) to find out what had already been developed to help older patients to self-manage many medicines at home. We found only very few interventions aimed at older patients with polypharmacy, only one of which targeted frail older patients. Most interventions sought to improve adherence and only a minority addressed a wider range of medicines self-management components, such as managing supply, checking the medicines received, monitoring side effects and escalating medicines-related concerns with the healthcare team. Our interview study revealed that, to most participants, managing multiple medicines felt like a complex job, and that complexity was increased by having to deal with a fragmented healthcare system where support and preparation were limited and staff roles often overlapped.  Drawing on the findings from the qualitative study and the literature review and informed by behaviour change theory we co-designed a complex intervention to support patients aged 65 or older, on 5 or more medicines, to self-manage their medicines safely. We used Experience Based Co-design (EBCD), a participatory method which has a short video of patients’ emotional touch points at its heart (Point of Care), to increase the relevance and acceptability of our intervention content and format. Involving staff in the co-design ensured that the complex intervention has potential to be developed in primary care despite the multiple constrains and pressures currently experienced in the UK national healthcare system. This intervention was developed working with frail older patients with polypharmacy, their family members and healthcare staff living or working in South and West Yorkshire, UK. The core stages of the study were conducted between April 2021 and April 2023 and were led by a multidisciplinary team including pharmacists with special interest in polypharmacy, frailty and multimorbidity, social researchers with experience in participatory methods and a co-applicant with lived experience of managing polypharmacy in older age at home.  A group of 3 older patients with polypharmacy and 2 family members with experience of supporting relatives with their multiple medicines joined the study as part of the Patient and Public Involvement Panel in May 2021. |
| --- | --- |
| **Item 2**  **Description:** Report the purpose of the intervention development process.  **Explanation:** Clearly describing the purpose of the intervention specifies what it sets out to achieve. The purpose may be informed by research priorities, for example those identified in systematic reviews, evidence gaps set out in practice guidance such as the National Institute for Health and Care Excellence, or specific prioritisation exercises such as those undertaken with patients and practitioners through the James Lind Alliance. | The research team aim was to develop a complex intervention, informed by Safety II theory and supported by a logic model, to make medicines self-management for older patients (aged 65 years or older) with polypharmacy (taking five or more medicines) safer and better (in terms of experience), by using participatory methods (EBCD). |
| **Item 3**  **Description:** Report the target population for the intervention development process.  **Explanation:** The target population is the population that will potentially benefit from the intervention—this may include patients, clinicians and/or members of the public. If the target population is clearly described, then readers will be able to understand the relevance of the intervention to their own research or practice. Health inequalities, gender and ethnicity are features of the target population that may be relevant to intervention development proceses. | The research team aim was to develop a complex intervention, informed by Safety II theory and supported by a logic model, to make medicines self-management for older patients (aged 65 years or older) with polypharmacy (taking five or more medicines) safer and better (in terms of experience), by using participatory methods (EBCD). |
| **Item 4**  **Description:** Report how any published intervention development approach contributed to the development process..  **Explanation:** Many formal intervention development approaches exist and are used to guide the intervention development process (eg, six steps in quality intervention development (6SQuID) or the person-based approach to intervention development). Where a formal intervention development approach is used, it is helpful to describe the process that was followed, including any deviations. More general approaches to intervention development also exist and have been categorised as follows: target population-centred intervention development; evidence-based and theory-based intervention development; partnership intervention development; implementation-based intervention development; efficacy-based intervention development; step-based or phased-based intervention development; and intervention-specific intervention development. These approaches do not always have specific guidance that describe their use. Nevertheless, it is helpful to give a rich description of how any published approach was operationalised. | The intervention development followed by the research team drew on how both Fylan et al. 2021 and Silcock et al. 2023 adapted EBCD, an approach more commonly used for quality improvement in healthcare settings, to design complex interventions, part of a multistage research study. |
| **Item 5**  **Description:** Report how evidence from different sources informed the intervention development process.  **Explanation:** Intervention development is often based on published evidence and/or primary data that have been collected to inform the intervention development process. It is useful to describe and reference all forms of evidence and data that have informed the development of the intervention because evidence bases can change rapidly, and to explain the manner in which the evidence and/or data were used. Understanding what evidence was and was not available at the time of intervention development can help readers to assess transferability to their current situation. | Our intervention development process was informed by behaviour change theory. We wanted someone (in our case a patient of a family carer) to do something differently to make medicines self-management at home safer and a ‘better experience’ for the ones involved, taking into account the constrains in the system.  Informed by Safety II, an approach that acknowledges patients’ roles in maintaining complex healthcare systems safe, we were interested in what patients already do right to keep safe around their medicines. Previous research, in fact, highlighted that patients and their family members can monitor their adherence, anticipate problems when their medicines or changed or respond to adverse events by escalating concerns with their care team. To improve medicines management experiences, we opted for a participatory approach. Experiences Based Co-Design (EBCD) was chosen among other methods because of the role played by people experiences in improving care and because it was also successfully adopted to develop complex interventions with similar populations and as part of multisite research study. |
| **Item 6**  **Description:** Report how/if existing published theory informed the intervention development process.  **Explanation:** Reporting whether and how theory informed the intervention development process aids the reader’s understanding of the theoretical rationale that underpins the intervention. Although not mentioned in the e-Delphi or consensus meeting, it became increasingly apparent through the development of our guidance that this item could relate to either existing published theory or programme theory. | Evidence from multiple sources informed the development process, including evidence from the literature, data collected interviewing patients and healthcare staff, notes from discussion at EBCD meetings and workshops, notes from meetings with the research team and the members of the Patient Advisory Group (PPI). List of type of evidence involved at each stage is reported below.  **Preparatory work**   - Review of evidence – data extracted from interventions retained - Experiences of patients and healthcare staff – qualitative interview study transcripts   **Priority setting**   - Priority setting meetings with patients and families – notes from discussion - Priority setting meetings with healthcare staff - notes from discussion - Joint staff, patients and families meeting - notes from discussion   **Identifying solutions**   - Co-design workshops - notes from discussion, Jamboard notes - Final event - notes from discussion   **Intervention prototyping**   - Staff focus groups – transcriptions of recordings and notes - Patients and families focus groups - transcriptions of recordings and notes   **Ongoing feedback**   - PPI ongoing feedback – notes from meetings and from one to one conversations, written feedback sent by email - Research Team Meeting notes, notes from conversations with patient co-applicant, written feedback sent by email |
| **Item 7**  **Description:** Report any use of components from an existing intervention in the current intervention development process.  **Explanation:** Some interventions are developed with components that have been adopted from existing interventions. Clearly identifying components that have been adopted or adapted and acknowledging their original source helps the reader to understand and distinguish between the novel and adopted components of the new intervention. | Not applicable |
| **Item 8**  **Description:** Report any guiding principles, people or factors that were prioritised when making decisions during the intervention development process.  **Explanation:**  Reporting any guiding principles that governed the development of the intervention will help the reader to understand the authors’ reasoning behind the decisions that were made. Guiding principles specify the core objectives and features of the desired intervention. These could include prioritising patient preferences over clinician preferences, providing an engaging experience for patients, minimising the cost of delivering the intervention, or maximising the potential for the intervention to be scaled up. | Four principles guided the team decision making:   1. Give priority to patients’ views   In case of conflicting views on directions to take for development, priority was given to patients’ suggestions. In case this was not possible, patient views were embedded in ideas for adaptations, alternative versions or further development. When for example the intervention prototype was taking the shape of an app or web app the voice of the patient co-applicant was loud and clear that this would have excluded a part of the older population. The team made the decision to develop the same components in two different ways of delivery: online and face to face.   1. Learn from what works   Safety II approach informed the way the research team looked at the data. Priority in data analysis and in the co-design of the prototype was given to actions and initiatives patients took to keep safe and in control of their medicines. The online version of our interventions includes a collection of tips, ideas patients tried and find helpful and wanted to share with others and things to watch out for while trying. Pharmacists in our team supervised all content in the materials to ensure safety.   1. Prevent or reduce burden   Patients and public involvement influenced the way the team looked at patient experiences of medicines management and sensitised them to the impact of medicines burden on daily life. Guiding principle of intervention development was to only include solutions that potentially contributed to make medicines management easier.   1. Patient centred care   People’s circumstances are different. Guiding principle was that the intervention should suggest tools and strategies and encourage people to try and adopt only the ones they felt appropriate |
| **Item 9**  **Description:** Report how stakeholders contributed to the intervention development process.  **Explanation:** Potential stakeholders can include patient and community representatives, local and national policy makers, healthcare providers, and those paying for or commissioning healthcare. Each of these groups may influence the intervention development process in different ways. Specifying how differing groups of stakeholders contributed to the intervention development process helps the reader to understand how stakeholders were involved and the degree of influence they had on the overall process. Further details on how to integrate stakeholder contributions within intervention reporting are available. | - A patient with lived experience of managing multiple medicines in older age was co-applicant and co-author in this study. - An advisory group of older people taking multiple medicines or with experience of supporting family members (PPI) played a key role in both the preliminary, the development and the prototyping stage of this research. - PPI contributed to identify key words to run searches at the start of the literature review. They also contributed to expand the restrictive definitions of medicines-self management encountered in the literature by co-writing a new definition, to embrace the wide range of skills and traits required to manage many medicines safely and with confidence. - PPI were involved in early data analysis in the interview study of patients and staff. - PPI helped to shape the chapters and the narrative of the ‘trigger film’, used in EBCD. - PPI advised on how to set up and run face to face meetings with patients and families and one of them take part as extra support and note taker. - PPI also advised on how to improve participation in online meeting once meeting face to face was no longer possible due to Covid-19 restrictions. - Two PPI members joined the co-design teams. - PPI gave feedback on each of the multiple prototype iterations, shaping the direction of the development at each step, including decisions around colour patterns and graphic design. - PPI reviewed all the scripts of patient led videos and all text in the online version of the prototype - Some PPI members took part in producing materials for the online version, working with the media partner. Some acted as ‘patient’ characters and give their voice and their face to I Manage My Meds.   Their ongoing involvement was essential in “setting the tone” of the intervention developing tools which people from the same population will potentially find both relevant and respectful. |
| **Item 10**  **Description:** Report how the intervention changed in content and format from the start of the intervention development process.  **Explanation:** Due to the iterative nature of intervention development, the intervention that is defined at the end of the development process can often be quite different from the one that was initially planned. Describing these changes and their rationale enhances understanding and enables other intervention developers to learn from this experience. For example, it may be that some intervention components were considered but ultimately discarded due to complexity or expense of delivery. | The intervention development started from a shared list of priorities, agreed by patients, families and healthcare professionals. Mixed co-design teams worked to transform the priorities into ideas for solutions to improve polypharmacy self-management for older people at home. Three ideas for solutions were brought forward for refinement. The research team merged overlapping elements and retained only components with potential to enhance safety in medicines management. To reduce complexity, the team also decided to condense the three refined solutions into a five components complex intervention. Following co-design participants and PPI preferences, the early prototype was designed in two different delivery modes. The first mode chosen was as online platform, giving access to tools and videos tutorials in patient’s perspective, that people could access directly. The second delivery mode chosen was a face-to-face support programme delivered by ‘previously trained’ expert patients.  The branding, the scripts for the videos, the combination of text, images and downloadable tools in the online version were refined by the research team working on iterative feedback received by multiple stakeholders, such as patients, families and healthcare professionals not previously involved in the research and experts in the care of older patients with polypharmacy.  An occupational psychologist helped to give structure and theoretical underpinning to the prototype, drawing om behaviour change techniques. A professional graphic designed the logo and the branding guidelines for the prototype. A media team created the website which is hosting the online version I Manage My Meds. |
| **Item 11**  **Description:** Report any changes to interventions required or likely to be required for subgroups.  **Explanation:** Specifying any changes that the intervention development team perceive are required for the intervention to be delivered or tailored to specific subgroups enables readers to understand the applicability of the intervention to their target population or context. These changes could include changes to personnel delivering the intervention, to the content of the intervention or to the mode of delivery of the intervention. | Adaptations concluded include an easy read version of online version and an Urdu translation of both the easy read and the full version of the online intervention. |
| **Item 12**  **Description:** Report important uncertainties at the end of the intervention development process.  **Explanation:** Intervention development is frequently an iterative process. The conclusion of the initial phase of intervention development does not necessarily mean that all uncertainties have been addressed. It is helpful to list remaining uncertainties such as the intervention intensity, mode of delivery, materials, procedures or type of location that the intervention is most suitable for. This can guide other researchers to potential future areas of research and practitioners about uncertainties relevant to their healthcare context. | Face to face version acceptability and further adaptations needed for that to be implemented in primary care will need further exploration. |
| **Item 13**  **Description:** Follow TIDieR guidance when describing the developed intervention.  **Explanation:**  Interventions have been poorly reported for a number of years. In response to this, internationally recognised guidance has been published to support the high-quality reporting of healthcare interventions[^5^](https://bmjopen.bmj.com/content/10/4/e033516#ref-5) and public health interventions.[^13^](https://bmjopen.bmj.com/content/10/4/e033516#ref-13) This guidance should therefore be followed when describing a developed intervention. | TIDier Guidance was used to describe the intervention and reported in Appendix 1. |
| **Item 14**  **Description:**  Report the intervention development process in an open access format.  **Explanation:**  Unless reports of intervention development are available, people considering using an intervention cannot understand the process that was undertaken and make a judgement about its appropriateness to their context. It also limits cumulative learning about intervention development methodology and observed consequences at later evaluation, translation and implementation stages. Reporting intervention development in an open access (gold or green) publishing format increases the accessibility and visibility of intervention development research and makes it more likely to be read and used. Potential platforms for open access publication of intervention development include open access journal publications, freely accessible funder reports or a study web page that details the intervention development process. | Article describing intervention development will be submitted to open access journal. |
